# Occupational safety and health status of medical laboratories in Kajiado County, Kenya

**DOI:** 10.11604/pamj.2018.29.65.12578

**Published:** 2018-01-23

**Authors:** Fridah Ntinyari Tait, Charles Mburu, Joseph Gikunju

**Affiliations:** 1 Institute of Energy and Environmental Technology, Jomo Kenyatta University of Agriculture and Technology, Kenya; 2 Institute of Energy and Environmental Technology, Jomo Kenyatta University of Agriculture and Technology, Kenya; 3 College of Health Sciences, Jomo Kenyatta University of Agriculture and Technology, Kenya

**Keywords:** Occupational safety and health, hazards, medical laboratories, Kajiado

## Abstract

**Introduction:**

Despite the increasing interest in Occupational Safety and Health (OSH), seldom studies are available on OSH in medical laboratories from developing countries in general although a high number of injuries occur without proper documentation. It is estimated that every day 6,300 people die as a result of occupational accidents or work-related diseases resulting in over 2.3 million deaths per year. Medical laboratories handle a wide range of materials, potentially dangerous pathogenic agents and exposes health workers to numerous potential hazards. This study evaluated the status of OSH in medical laboratories in Kajiado County, Kenya. The objectives included establishment of biological, chemical and physical hazards; reviewing medical laboratories control measures; and enumerating factors hindering implementation of good practices in OSH.

**Methods:**

This was a cross-sectional descriptive study research design. Observation check lists, interview schedules and structured questionnaires were used. The study was carried out in 108 medical laboratories among 204 sampled respondents. Data was analysed using statistical package for social science (SPSS) 20 software.

**Results:**

The commonest type of hazards in medical laboratories include; bacteria (80%) for Biological hazards; handling un-labelled and un-marked chemicals (38.2%) for chemical hazards; and laboratory equipment's dangerously placed (49.5%) for Physical hazards. According to Pearson's Product Moment Correlation analysis, not-wearing personal protective equipment's was statistically associated with exposure to hazards. Individual control measures were statistically significant at 0.01 significance level. Only 65.1% of the factors influencing implementation of OSH in medical laboratories were identified.

**Conclusion:**

Training has the highest contribution to good OSH practices.

## Introduction

In Kenya, the status of Occupational Safety and Health (OSH) conditions has been an issue of growing importance over time. OSH issues in Kenya can be traced back to 1951's Factories' Ordnance Act, which later became the Factories Act Cap 514 laws of Kenya. In 2004, the Government gazetted a subsidiary legislation titled “Factory and Other Places of Work (Safety and Health Committee) Rules, 2004” Legal Notice No. 31 that created Safety Committees in factories and other places of work that regularly employed more than 20 employees. These committees were tasked with the responsibility for overseeing OSH implementation, and performing safety audits. However, shortfalls remained with reports that more than half of the work related accidents and injuries went unreported or unattended, necessitating the birth of Occupational Safety and Health Act (OSHA) 2007 intended to give a more elaborate approach to OSH issues [1]. Currently many people are being employed in medical laboratories than in the past years due to the growing number of clinical investigations available and therefore an increased need for technicians and technologists to perform the tests. These staff members are exposed to a broad variety of hazards linked with the equipment they use and the methods they employ in the line of their duty. The dangers associated with a substance process may not be realized until some unforeseen illness, accident, or perhaps death happen. Health care workers are known to be at a higher risk of infection from blood-borne pathogens than the general population [2]. Those most at risk are those whose activities entail exposure to blood and body fluids. Important blood-borne pathogens in this regard include Hepatitis B (HBV), Hepatitis C (HCV) and HIV/AIDS according [3]. It is estimated that every day 6,300 people die as a result of occupational accidents or work-related diseases resulting in over 2.3 million deaths per year. This is on the background of over 337 million on-the-job accidents annually resulting from poor occupational safety and health practices [4]. However, the rate of related injuries (both reported and non-reported) is believed to be much higher and no data specific to OSH in medical laboratories has been documented. Medical laboratories handle a wide range of materials and a large number of potentially dangerous pathogenic agents. There is a lesser possibility that certain samples contain pathogenic agents for example mycobacterium tuberculosis in sputum specimens, blood borne pathogens e.g. Hepatitis B, C & HIV, salmonellosis, brucellosis and many others. Sharps injuries contribute over 30% of new cases of Hepatitis B Virus and 2.5% of annual infections of HIV among health care workers in Sub-Saharan Africa [5]. In addition to handling of ineffective material medical laboratories use chemical agents, gases and solvents that constitute non-biological hazards. These agents can be explosive, flammable or toxic and fires, gassings and explosions and can occur in the labs [6, 7]. Laboratories inherently have significant physical hazards which include electrical hazards, handling sharps, ergonomic hazards associated with manual material handling and equipment use [8]. Noise from equipment such as centrifuge could also be detrimental to the ears. The workers are also faced with ergonomic hazards such as sitting on very high chairs which can cause musco-skeletal disorder due to prolonged standing and repetitive tasks. The aim of the Kenyan Occupational Safety and Health Act (OSHA) is to secure the safety, health and welfare of people at work and to protect those not at work from risks to their safety and health arising from, or in connection with, the activities of people at work [9]. This study therefore sought to establish the different biological, chemical and physical hazards that medical workers are exposed to; reviewing medical laboratories control measures that are put in place; and enumerating the different factors that hinder implementation of good practice in OSH for medical laboratories.

## Methods

**Study site**: The study was conducted at all health facilities with medical laboratories, Kajiado County, Kenya. Kajiado County is located in the Rift valley part of Kenya [10]. It has a Total Population of approximately 800,000 households covering an area of 21,902.9 Sq. kilometers. It has 118 medical laboratories with 250 registered medical laboratory technicians and technologists. The laboratory workers in these laboratories work for long hours and in most cases, will get a lone worker in the laboratory due understaffing. The medical laboratories are normally located in the furthest corners of the facility, often next to the wash rooms and are the smallest rooms with minimal ventilation and space.

**Study design**: It was a cross-sectional study in which semi-structured key-informant individual interviews were conducted over a one month period. Individual interviews help to collect insightful descriptions from participants [11]. Purposive sampling method was used and for the purpose of this study all registered medical laboratories in Kajiado County formed the target population of the study. Approximately 250 questionnaires and consent forms were distributed to the health workers. Of these, 214 (85.6%) were returned, signed by participants. Institutional consent was also sought among 118 health facilities with medical laboratories. Of these, 108 (91.53%) duly signed copies of the consent were returned. The medical laboratory staff not registered under the Kenya Medical Laboratories act were excluded from the study including those who declined to sign consent forms (36 medical laboratory staff). Further 10 cases data were incomplete. The final sample was composed of 204 (82%) medical laboratory staff. This sampled staff completed the questionnaires that had variables with combined close and open responses.

**Instruments**: All the questionnaires were self-reported and were completed by the participants with the aide and observation of a trained researcher about all aspects of the questionnaires. Each interview began with obtaining consent from the participant, explanation of the study followed by the participants filling the questionnaires. Participants were assured of the confidentiality of their responses. Observation checklist was also used. The questionnaire consisted of 24 questions that included a number of demographic variables; biological, chemical and physical hazards variables; control measures variables; and variables to establish the factors that hinder good OSH practices in medical laboratories with combined close and open responses. The observation checklist had 15 variables to assess the status of OSH in medical laboratories. Once a questionnaire is finalized, it should be tried out in the field. A pre-test for the data collection tools was conducted to ensure reliability. Split half method was used to determine the reliability of the instruments. In order to ensure validity, all the researchers participated in data collection to ensure triangulation by having a team research approach. At the same time triangulation was done by comparing data to already existing literature on Occupational Safety and Health [12].

**Statistical Analysis**: The collected data was entered into Epi data version 3.1 software, cleaned with Stata version 13 and were analyzed using the Statistical Package For the Social Sciences, SPSS Inc., Chicago, IL (SPSS version 20). Thematic content analysis, a valued method for analyzing qualitative data was also used. Bivariate, multivariate and logistic regression model were the methods used to analyse the quantitative part of the data using SPSS. A Chi-square was used to test statistical significance [12]. On factors that hinder implementation of good practice of Occupational Safety and Health practices findings, Pearson's Product Moment Correlation Coefficient (PPMCC) analysis for the factors was conducted: this is to examine the strength of the relationship between the variables by examining the extent the statistical significance of the relationship and the extent of the correlation coefficient. This correlation coefficient (usually represented by the letter r) normally take on any value between -1 and +1 [12]. A value of +1 represents a perfect positive correlation while a value of -1 represents a perfect negative correlation. A value of 0 means the variables are perfectly independent. As outlined, if this probability (p) is less than 0.01 (p < 0.01) or 0.05 (p < 0.05) then it is considered statistically significant. If the probability is greater than 0.01 (p > 0.01) or 0.05 (p > 0.05) then the relationship is not statistically significant [13]. Findings were presented in Tables and figures based on the major research objectives.

**Ethical issues**: Permission was obtained from the Institute of Energy and Environmental Technology of Jomo Kenyatta University and also from County Department of Health Services, Kajiado. Consent was also obtained from each study participant. Confidentiality, autonomy, respects and dignity of all the participants was strictly observed throughout the study. Additionally participants were assured of their rights to decline participating in the study and also not to answer questions they felt uncomfortable with. The participants were also assured that there will be no harm, prejudice, malice or any form of danger should they wish not to participate in the study.

## Results

**Social demographic data**: Most (51.5%) of the respondents were females and the majority (60.3%) of respondents were aged 19-30 years with a combined mean age of 30.1 years ± 7.1 SD. The respondents were mostly of Diploma level of education (78.43%) and close to one-half of them had 2-5 years of experience (Table 1).

**Table 1:** Socio-demographic characteristics of respondents

Characteristics	Frequency	Percentage
**Age group (years)**		
19-30 Years	123	60.3%
31-42 Years	66	32.4%
43 Years and above	15	7.4%
**Sex**		
Male	99	48.5%
Female	105	51.5%
**Education Level**		
Diploma	160	78.43%
Higher Diploma	21	10.29%
Degree	20	9.8%
Masters	3	1.47%
**Years of experience**		
1 year and below	9	4.4%
2-5 years	102	50.0%
6-10 years	72	35.3%
11 years and above	21	10.3%

### Biological, chemical and physical hazards

**Biological hazards**: The study identified biological hazards in Phlebotomy, specimen processing area, waiting bay and at the Slide preparation areas. As shown in Table 2, 80% of the respondents reported exposure to Bacteria, 47% exposure to Parasites, 17% exposure to fungi, while only 8% reported exposure to viral vectors. On average, 65.6% of the respondents reported to have been exposed to at least a type of biological hazard. Further analysis indicates that there were significant correlations between; age with exposure to bacteria (r = -0.166, 0.05 significance level) and parasites (-0.157, 0.01 significance level); Education with exposure to bacteria (r = 0.160, 0.05 significance level); Years of experience had correlations with exposure to fungi (r = -0.561) and Viral vectors (r = -0.342) at 0.01 significance level respectively.

**Table 2:** Exposure to biological hazards by laboratory staff working in medical laboratories in Kajiado County, Kenya (n = 204)

		Biological Hazard (n=204)			
Characteristic	Category	Bacteria	Parasite	Fungi	Viral
		n (%)	n (%)	n (%)	n (%)
**Overall**	Total	164(80%)	95(47%)	35(17%)	17(8%)
**Sex**	Male	79(48%)	48 (51%)	20 (57%)	14(82%)
	Female	85 (52%)	47 (49%)	15 (43%)	3 (18%)
**Education**	Diploma	122 (74%)	72(76%)	29 (83%)	16 (94%)
	Higher Diploma	21(13%)	14 (15%)	4 (11%)	-
	Degree	19 (12%)	8 (8%)	1 (3%)	1 (6%)
	Masters	2 (1%)	1 (1%)	1 (3%)	-
**Age**	19-30 Years	92 (56%)	51 (54%)	22 (63%)	14 (82%)
	31-42 Years	59 (36%)	32 (34%)	9 (26%)	2 (12%)
	43 and above years	13 (8%)	12 (13%)	4 (11%)	1 (6%)
**Years of experience**	1 Year and below	9(5%)	5 (5%)	1(3%)	-
	2-5 years	75 (46%)	41 (43%)	20(57%)	11(65%)
	6-10 years	62 (38%)	40 (42%)	13(37%)	4(24%)
	11 years and above	18 (11%)	9 (9%)	1(3%)	2(12%)

**Figure 1:**
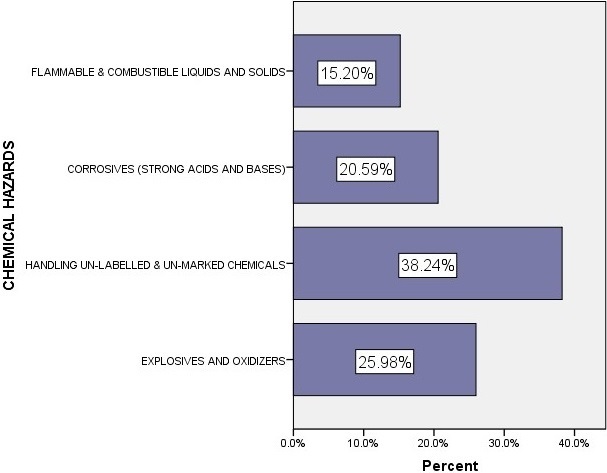
Hespondents exposure to Chemical hazard

**Chemical hazards**: The study findings indicate that 38.24% of the respondents handle un-marked and un-labelled chemicals with only 15.2% exposed to flammable & combustible liquids, and flammable solids as shown in the Figure 1. Further analysis indicated that there is no significant relationship between demographic factors and exposure to Chemical hazards (p = not significant). The study found out that 23% of respondents observed without personal protective equipment (60% did not wear PPEs) reported having been exposed to Chemical hazards. Further analysis showed that not wearing personal protective equipment's was statistically associated with exposure to chemical hazards (p = 0.0067) posing a risk factor for the health workers. All health facilities surveyed had different chemicals being used with 70% having chemical which were un-labelled, 61% of laboratories had chemicals classified as explosives, oxidizers & organic peroxides, 69% had corrosives and 52% had flammable and combustible liquids & solids. It was further reported that there was an acute shortage of antiseptics in most of the health facilities and that supply of most laboratory chemicals has on inconsistent rate.

**Figure 2:**
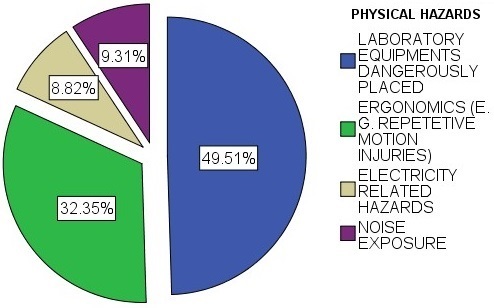
Pie chart showing respondents exposure to physical hazards

**Physical hazards**: Generally, respondents indicated the major type of physical hazard was laboratory equipment's dangerously placed (49.51%) followed by ergonomics (32.35%) as shown in Figure 2. The study further found a weak negative correlation (r = -.043) between exposures to physical hazards with gender and a weak positive correlation (r = 0.065) between level of education and the respondent having exposed to physical hazards at 0.05 significance level. Health workers of 19-30 years had higher exposures on all forms of physical hazards reported on, though further analysis indicated a very weak correlation (r = -0.084) between age and exposure to physical hazards at 0.05 significance level. Years of experience had a weak negative correlation (r = -0.013) at 0.05 significance level. The research grouped the physical hazards observed into Equipment related hazards, laboratory environment hazards and compressed gases and pressure related hazards. From the findings, 57% of the laboratories had their workers exposed to electrical shocks by wearing rings, watches and other jewellery when working around electrical appliances, while 51% of the laboratories had poor disposal mechanisms especially for broken glassware. Only 80% of the facilities were observed to have their electrical equipment connected to backup power cut-off. Further, it was observed that 51% of the laboratories had their safe working pressure unmarked, 50% did not have fire extinguishers installed, and 42% had their gas cylinders not suitably located. Most laboratories (73% and 72% respectively) had their laboratories having warning restriction signs, secured pressurized gas cylinders, with 62% having no warning hazard signs and their pressure vessels periodically not examined respectively. Findings indicate that 100% of medical laboratories workers were exposed to prolonged viewing to a microscope due to lack of adjustable chairs and microscopes without affixed video cameras' which can cause problems with the neck and shoulders as well as eyestrain. They were observed to use pipettes that are thumb-operated that can lead to soreness and eventual repetitive use injury instead of trigger operated and/or electric pipette pumps. Further, 74% of the health facilities are exposing their health workers to prolonged standing at the laboratory benches instead of use of a stool that can be adjusted to a proper height, 48% of the laboratories with inadequate lighting, 94% of laboratories lacking hearing protective devices especially when operating the centrifuges and 56% of the laboratories having no restriction signs on bio-hazardous areas. Though nose masks and protective gloves were 100% provided in all the laboratories, only 47% of the laboratories were observed to have their health workers fully utilize nose masks and 58% use of protective gloves as personal protective equipment. In 28% of the laboratories the pressurized gas cylinders were not secured while in 42% of the laboratories.

**Control measures to mitigate the OSH hazards**: The researchers made an inquiry into the occupational measures in place to control occupational safety and health hazards. The research grouped control measures into the health facility measures, individual measures and handwashing measures that were in place to control occupational safety and health hazards as shown in Table 3. According to the correlation findings in Table 4, the individual protective control measures had strong correlation with each other and that they are statistically significant at level 0.01. These means that a number of health workers are cognitive of taking necessary precautions for health and safety. According to the correlation findings (Table 5), the hand washing practices had strong correlation with each other and that they are statistically significant at level 0.05. These findings are largely similar to other previous studies conducted in low and middle income countries.

**Table 3:** Control measures for occupational safety and health employed by laboratory staff working in medical laboratories, Kajiado County, Kenya

Occupational safety and health control measures	Frequency (N=204)	
Control measures provided by employers	N	(%)
Medical facility has first aid box	75	36.8%
Medical facility has proper waste disposal equipment	189	92.6%
Provision of Antiseptics	21	10.3%
Medical facility has chemical hoods	39	19.1%
Medical facility has chemical hygiene plan	51	25.0%
**Individual protective measures**		
Health workers received HIV screening	177	87%
Provided with post exposure prophylaxis	147	72%
Provided with Hepatitis A vaccination	73	36%
Provided with Hepatitis B vaccination	167	82%
Provided with BCG vaccination	194	95%
Provision of personal protective equipment’s	122	60%
**Hand washing Practices**		
Before and after laboratory procedures	155	76%
After removing the gloves	112	55%
After handling soiled materials	118	58%
Before and after handling clients/ each patient	147	72%
After handling biological samples and other hazardous materials	126	62%

**Table 4:** Individual protective measures correlations for medical laboratory staff working in Kajiado County, Kenya

		Health Workers Received HIV Screening	Provided with Post Exposure Prophylaxis	Provided with Hepatitis A Vaccination	Provided with Hepatitis B Vaccination	Provision of Personal Protective Equipment	Use of Disinfectants
Health Workers Received HIV Screening	Pearson Correlation	1	-0.018	0.020	-0.109	0.020	0.013
	Sig. (2-tailed)	-	0.803	0.777	0.122	0.771	0.856
	N	204	204	204	204	204	204
Provided with Post Exposure Prophylaxis	Pearson Correlation	-0.018	1	0.009	0.075	0.084	-0.016
	Sig. (2-tailed)	0.803	-	0.898	0.283	0.234	0.819
	N	204	204	204	204	204	204
Provided with Hepatitis A Vaccination	Pearson Correlation	0.020	0.009	1	-0.047	-0.069	0.808^**^
	Sig. (2-tailed)	0.777	0.898	-	0.507	0.329	0.000
	N	204	204	204	204	204	204
Provided With Hepatitis B Vaccination	Pearson Correlation	-0.109	0.075	-0.047	1	-0.040	-0.050
	Sig. (2-tailed)	0.122	0.283	0.507	-	0.565	0.479
	N	204	204	204	204	204	204
Provision Of Personal Protective Equipment	Pearson Correlation	0.020	0.084	-0.069	-0.040	1	-0.044
	Sig. (2-tailed)	0.771	0.234	0.329	0.565	-	0.532
	N	204	204	204	204	204	204
Use Of Disinfectants	Pearson Correlation	0.013	-0.016	0.808^**^	-0.050	-0.044	1
	Sig. (2-tailed)	0.856	0.819	0.000	0.479	0.532	-
	N	204	204	204	204	204	204

** Correlation is significant at the 0.01 level (2-tailed)

**Table 5:** Hygienic hand disinfection correlations for medical laboratory staff working in medical laboratories, Kajiado County, Kenya

		Before and After Every Laboratory Procedure	After Removing the Gloves	After Handling Soiled Materials	Before and After Handling Patients	After Handling Biomaterials and Other Hazardous Materials
Before and After Every Laboratory Procedure	Pearson Correlation	1	-0.134	0.782^**^	-0.078	0.608^**^
	Sig. (2-Tailed)	-	0.056	0.000	0.267	0.000
	N	204	204	204	204	204
After Removing the Gloves	Pearson Correlation	-0.134	1	-0.097	-0.051	-0.103
	Sig. (2-Tailed)	0.056	-	0.169	0.465	0.144
	N	204	204	204	204	204
After Handling Soiled Materials	Pearson Correlation	0.782^**^	-0.097	1	-0.079	0.536^**^
	Sig. (2-Tailed)	0.000	0.169	-	0.259	0.000
	N	204	204	204	204	204
Before and After Handling Patients	Pearson Correlation	-0.078	-0.051	-0.079	1	-0.010
	Sig. (2-Tailed)	0.267	0.465	0.259	-	0.883
	N	204	204	204	204	204
After Handling Biomaterials and Other Hazardous Materials	Pearson Correlation	0.608^**^	-0.103	0.536^**^	-0.010	1
	Sig. (2-Tailed)	0.000	0.144	0.000	0.883	-
	N	204	204	204	204	204

** Correlation is significant at the 0.01 level (2-tailed)

**Factors that hinder implementation of good OSH practices**: The study sought to determine the factors that hinder implementation of good practice of Occupational Safety and Health practices. Pearson's Product Moment Correlation Coefficient (PPMCC) analysis for the factors was conducted: This was to examine the strength of the relationship between the variables (design of lab, ignorance level, lack of Personal Protective Equipment(s) PPEs, inadequate resources, inadequate Occupational Safety Health (OSH) training, lack of OSH policy, negative attitude and poor ergonomics); by examining the extent the statistical significance of the relationship and the extent of the correlation coefficient. According to the correlation the findings in Table 6, the factors had strong positive correlation with each other and that they are statistically significant at level 0.01. From the regression summary results of the factors, R = 0.727a (predictors: (constant), poor ergonomics, inadequate training, lack PPE, ignorance, lab design, no policy, negative attitude, inadequate resources), the R Square is 0.651 i.e. 65.1% of the factors were identified in the study. The remaining 34.9% indicated that there are other factors which influenced implementation of good practice of Occupational Safety and Health practices in health facilities which were not identified in the study. Coefficient analysis of all the factors indicated that training has the highest contribution to good practice of occupational safety and health practices with a unit increase of OSH training leading to increases in each of the other factors.

**Table 6:** Correlation matrix of factors hindering occupational safety and health among laboratory staff working in medical laboratories, Kajiado County, Kenya

		design of lab	Ignorance	PPEs	Resources	Training	Policy	Attitude	Ergonomics
Poor design of lab	Pearson Correlation	1							
	Sig, (2 –tailed)								
	N	81							
Ignorance/lack of awareness	Pearson Correlation	0.724^**^	1						
	Sig, (2 –tailed)	0.000							
	N	54	54						
Lack of PPE(s)	Pearson Correlation	0.875^**^	0.721^**^	1					
	Sig, (2 –tailed)	0.000	0.000	-					
	N	18	18	18					
Inadequate resources/ infrastructure	Pearson Correlation	0.612^**^	0.861^**^	0.832^**^	1				
	Sig, (2 –tailed)	0.000	0.000	0.000	-				
	N	84	84	84	84				
Inadequate training on OHS	Pearson Correlation	0.812^**^	0.762^**^	0.839^**^	0.869^**^	1			
	Sig, (2 –tailed)	0.000	0.000	0.000	0.000	-			
	N	60	60	60	60	60			
No policy on OHS	Pearson Correlation	0.362^**^	0.422^**^	0.534^**^	.883^**^	0.896^**^	1		
	Sig, (2 –tailed)	0.000	0.000	0.000	0.000	0.000	-		
	N	71	71	71	71	71	71		
Negative attitude on OHS	Pearson Correlation	0.763^**^	0.761^**^	0.837^**^	0.684^**^	0.899^**^	0.842^**^	1	
	Sig, (2 –tailed)	0.000	0.000	0.000	0.000	0.000	0.000	-	
	N	96	96	96	96	96	96	96	
Poor ergonomics	Pearson Correlation	0.843^**^	0.867^**^	0.697^**^	0.569^**^	0.846^**^	0.814^**^	0.781^**^	1
	Sig, (2 –tailed)	0.000	0.000	0.000	0.000	0.000	0.000	0.000	-
	N	12	12	12	12	12	12	12	12

** Correlation is significant at the 0.01 level (2-tailed)

## Discussion

This study focused more on specific category of health care workers hence the disparity in the socio demographic data and further disagrees with the outcomes on occupational health hazards study among 200 respondents (health care workers) who worked in 8 major health facilities in Kampala, Uganda whose results indicated male respondents were 28.5% while female respondents were 71.5% [4]. Our results are comparable with those on status of occupational safety among health service providers in Tanzania which indicated that majority of the 430 respondents did not have post graduate degree training and that none had received training on Occupational Safety and Health as a profession [14]. The mean age of respondents in the present study was 30.1 and SD was 7.1. Our results are comparable with those on knowledge, attitudes and practices of laboratory safety at University of Port Ha-court teaching hospital, Nigeria, in which the respondents mean age was 35.3 and SD was 8.8 representing a youthful representation [15].

**Biological, chemical and physical hazards**: In the current study, at least 65.6% of the respondents reported to have been exposed to at least a type of biological hazard. 80%, 47%, 17% and 8% of the respondents reported to have had exposure to bacteria, Parasites, Fungi and Viruses respectively. The high percentages for exposure to bacteria is attributable to the fact that most bacterial habitats surrounding humans are either in digestion systems as normal flora or present as infection. In addition, biological hazards are present in various sources throughout the lab such as blood and body fluid, culture specimens, body tissues and cadavers, as well as other workers [15]. Our results are comparable with those on occupational health hazards among health workers in Kampala, Uganda whose findings indicated that majority of the respondents reported having exposure to biological hazards (39.5%) as compared to 31.5% who experienced non-biological hazards and also that not wearing necessary personal protective equipment (AOR = 2.34 (1.29-4.64), p = 0.006) is an independent predictors for experiencing a biological hazard [4]. Handling un-labelled or un-marked chemicals were the main chemical hazards affecting the workers of medical laboratories (38.24%) in this study. Our results are comparable with those on a survey of safety practices among hospital laboratories in Oromia regional state in Ethiopia which revealed that although there were lists of chemical records in all laboratories assessed, all chemicals were not labeled with full chemical information and it is unknown who labels some of the chemicals [16]. Laboratory equipment's dangerously placed (49.51%) and ergonomics related factors (32.35%) were the main physical hazards in this study. Our findings on laboratory equipment's dangerously placed are not in compliance with International Labour Organization recommendations that hazards and risks to workers' safety and health must be identified and assessed on an ongoing basis [17]. Our findings further indicated that all medical laboratories workers were exposed to prolonged standing while viewing a microscope, 94% of laboratories lacked hearing protective devices, and 60% of respondents did not wear any Personal Protective Equipment while in the medical laboratory. These percentages are relatively high compared to a study conducted in Pakistan which revealed that 46.2% of the laboratory technicians did not use any kind of personal protective equipment and that of a Turkish study where 91.3% and 87.4% of the participants used gloves and lab coats, respectively [18, 19]. Our findings further indicated a weak negative correlation (r = -0.043) between exposures to physical hazards with gender. This finding was consistent with the finding on the study on gender differences in occupational exposure patterns [20]. Our findings further indicate that 57% of the laboratories had their workers to electrical shocks by wearing rings, watches, and other jewellery when working around electrical appliances while only 20% of the facilities have their electrical equipment connected to backup power cut-off. Our results are comparable with those on occupational hazards in hospitals indicating that 23% of human error accidents in the work place are electrical related and may lead directly to internal and external burns, or gaseous embolism; and indirectly, in the form of burns or asphyxia produced by electrical fires or explosions, or injuries suffered in falls after electric shock [21].

**Control measures to mitigate the OSH hazards**: In the current study, the major control measures provided by the health facilities were training and supervising staff on occupational safety and health (98%), availing proper containers to dispose medical waste (92.6%) and first aid safety tools and equipment (36.8%). In addition, our findings indicate that 25% and 19.1% of the facilities have chemical hygiene plans and chemical hoods respectively. Our results further indicate that majority of laboratory workers had received HIV screening examination (87.0%) and 95.0% had received BCG vaccination. Regarding the hand washing practices, our results indicate that most laboratory staff washed their hands before and after every procedure (76%) and after handling soiled materials (58%). Seventy two percent of laboratory workers washed hands before and after handling clients while only 62% after handling samples and other hazardous materials. Our findings are comparable with other similar studies which established that using all the necessary personal protective equipment was associated with reduced exposure to hazards. Other similar findings report that most health facilities are provided with waste disposal facilities for the medical waste and apply simple measures like hand washing as control measures for occupational health hazards and further indicate that hand washing practices are not fully embraced in most health facilities. The proportion of health workers who reported washing hands after recommended procedures was lower than has been reported by previous studies. Our results are also comparable with study findings that majority of health workers in Uganda had been screened for HIV (97%) and 91.0% had received BCG vaccination [4, 22, 23]. In the current study nearly 90% of the respondents reported that their employers had not provided antiseptics in the medical laboratories. Our results are comparable with those of a study in Tanzania on status of occupational safety among health service providers, in which antiseptics were not equally available in the hospitals, due mainly to procurement problems and problems inherent in the supply chain for the drugs and other supplies in government hospitals [14]. These findings are also are in compliance with ILO's recommendations for occupational health and safety that hand washing or antiseptic use after glove removal is a key requirement in undertaking laboratory procedures and infection control and also Centre for Disease Control recommendations that if hands are not visibly soiled, an alcohol-based waterless agent may be used for routinely decontaminating hands [24, 25]. Our findings that 60% of the medical laboratories employees had not been provided with all the appropriate Personal Protective Equipment's by their employers were not in compliance with International Labour Organization's recommendation that provides that employers must assess tasks to identify potential worksite hazards and provide and ensure that workers use appropriate personal protective equipment (PPE) [24, 26]. Our findings are also supported by Hayden et al who reported that use of PPEs reduced acquisition of illnesses in hospital settings [27]. Other studies have reported that use and compliance with utilization of PPEs has for long been recognized as important infection control measure in the healthcare industry which should be emphasized to minimize exposure to occupational health hazards [22, 23].

**Factors that hinder implementation of good OSH practices**: In this study, regression results of factors which influenced implementation of good practice of Occupational safety and health practices in health facilities (34.9%) were not identified. Our results indicate that exposure to hazards were associated with poor design of the laboratory facility (39.7%), not wearing all the necessary personal protective equipment (34.8%), Ignorance among health workers (36.8%), Inadequate resources (41.2%), Inadequate training on Occupational Safety and Health (29.4%), No policy on OSH (34.8%), Negative attitude on OSH (55.9%) and Poor ergonomics (5.9%). Our findings also indicate that training alone has the highest contribution to good practice of occupational safety and health practices with a unit increase of OSH training leading to increases in each of the other factors. Past empirical studies were apparently in agreement with these findings [1, 15, 28] Our findings on training of staff on OSH are also comparable with those of a study that was carried out on the relationship between employees' perceptions of safety and organizational culture where insufficient safety training was the root cause of major accidents at the work place since employees did not have the knowledge and skills to identify potential hazards [26, 29]. In a similar study, organizations which emphasized on safety through training and other managerial practices observed an increase in safety compliance among their employees [15]. Other similar study further reported that employees who have received safety training will likely report less work-related injuries than their untrained counterparts [28]. Other studies have further established that training allowed employees to acquire greater competencies to manage their work, leading to enhancement of their occupational safety [30].

## Conclusion

This research concludes that bacteria is the commonest (80%) type of Biological hazards, handling un-labelled and un-marked chemicals (38.2%) is the commonest type of chemical hazards and laboratory equipment's dangerously placed (49.5%) formed the commonest type of Physical hazards at medical laboratories in Kajiado County. The most predisposed category of medical laboratory workers were young employees with between 2-5 years of experience. The research further concludes that not wearing personal protective equipment's was a major predisposing factor to exposure to hazards. There is lack of qualified personnel for OSH in medical laboratories and that OSH is given least priority in the health sector. The research further concludes that training on OSH among health workers be strengthened as it has the highest contribution to good practice of occupational safety and health practices. This study indicates that there is a shortage of health workers trained on occupational safety and health in medical laboratories in Kajiado.

### What is known about this topic

- The laboratory environment can be a hazardous place to work;
- Laboratory workers are exposed to numerous potential hazards including chemical, biological, physical and radioactive hazards, as well as musculoskeletal stresses;
- Risk assessment is the backbone of occupational health and safety for laboratories.

### What this study adds

- Specific focus on the various hazards medical laboratory workers are exposed to;
- Study findings elaborate on the importance for employers to strengthen their institutional mechanisms to minimise occupational safety and health related hazards.

## Competing interests

The authors declare no competing interest.
